# Tuberculous Abscess in the Pancreas

**Published:** 1847-04

**Authors:** 


*Tuberculous Abscess in the Pancreas.*—The rare occurrence of disease of the pancreas renders the following case interesting. The subject of the case was a young female, aged 25, who had for a considerable time been out of health. Four years ago she had suffered for about two months from severe pain at the lower part of the chest, extending through to the back, which prevented her from lying on her right side or on her back. After the subsidence of this attack she seems to have continued in tolerable health for nearly three years, at the end of which time she became affected with lassitude and debility, uneasiness in the chest and bilious vomiting. The vomiting would sometimes last four or six hours. From this time her skin slowly and gradually became jaundiced, and continued so to the date at which she came under M. Cruveilhier's care, who has recorded the case. She complained at this time chiefly of loss of appetite, and inability to lie on her back in bed unless propped up. Her tongue was moist, and digestion good; but her bowels were obstinately confined, so that she did not require to go to the closet oftener than once in four or five days. The evacuations possessed their natural colour. She had some uneasiness on pressure over the epigastrium. The margin of the liver could be felt below the cartilages of the ribs, and appeared slightly enlarged: some pain was caused by pressure over this organ. The urine was not high coloured, and formed no precipitate on the addition of nitric acid. She had now very little uneasiness in the chest; no cough, palpitation, or headache. Neither by auscultation nor by percussion could any thing abnormal be detected in the lungs or heart. Some slight remedies were made use of. In about a week she became feverish and generally ill, without any known cause: in the evening of the same day she vomited some bile. The vomiting continued next day, and was accompanied by loss of appetite, and severe pain at the pit of the stomach. The pulse was 100, small and feeble. The vomiting was almost incessant for the two following days, when she became extremely exhausted: the pain in the epigastrium was most severe. No evacuation from the bowels had taken place since the commencement of the attack. Tongue dry, furred, white on the dorsum, red at the tip. Pulse 90, small. She died exhausted the same evening.

The liver was found rather larger than natural; its right lobe firm in texture, and of a peculiar greyish green colour throughout. About two ounces of dark green bile were contained in the gall-bladder. The glands in the neighbourhood of the stomach were enlarged, softened, and infiltrated with a blackish matter. Those near the pancreas were also enlarged, and filled with tuberculous matter. The pancreas was about natural in size. Within its last half, or tail, was found an abscess, capable of holding a small hen's egg. It was filled with thick purulent matter, and its cavity was lined by a thick and tough, greyish, organised membrane, within and around which were numerous softened tubercles. External to the abscess the tissue of the pancreas was spread out, and appeared atrophied. The right half of the pancreas was dark coloured, but healthy in structure. The pancreatic duct was entire. Two small cretaceous masses of tubercles were found in the spleen, and two similar ones in the apex of the left lung. Traces of recent violent inflammation were found in the stomach, the duodenum, and for a short way down the jejunum.—*Ibid. from Gaz. des Hôpitaux.*

